# Glioma of Retina

**Published:** 1880-03

**Authors:** W. Cheatham


### GLIOMA OF RETINA.

By W. CHEATHAM, M.D.

This is not of very common occurrence. In 1879 I saw three cases, two of which I think worth reporting. Glioma is a species of the round cell sarcoma. It occurs in the brain, cranial nerves and retina. It springs from neuralgia connective tissue of nerve. For some time there was great doubt as to its malignancy, and especially as to its capability of spreading to distant organs. The possibility of such an occurrence has been proved by many cases. The disease may extend along the optic nerve to the brain; consequently an early enucleation should be advised. After the disease attacks the orbital tissue it extends quite rapidly. The causes are quite obscure. It is sometimes of traumatic origin. It is hereditary, as it sometimes attacks several members of the same family. Lerch and Sickel have both seen it in four children of the same family.

The prognosis is exceedingly grave. It very often returns; by many said to always return. It has been urged by many that it is useless to enucleate such an eye, as the disease is sure to return and an early death result.

When I am consulted in reference to such a trouble I usually advise the parents in the following manner: The eye should be removed immediately. If it is enucleated before the disease breaks through the coats of the eye, there are some few chances of it not returning. If it should return, it gives great hopes of it attacking some internal organ, and resulting in an early fatal termination. If left as it is, it will break through the coats of the eye and attack all surrounding tissue, and very likely almost eat off the head of the little patient before death.

Such a course would be exceedingly disagreeable and painful to both patient and relatives. In other words, I believe, should I be a subject of such a disease, and even if I were sure that an anucleation could not stop the disease, but would drive it to some vital organ and thus shorten my days of suffering, I would say, Take my eye out.

The first case reported to me a few months before the disease broke through the sclerotic or sclero-corneal junction. I then advised an early enucleation. In six or seven weeks the globe ruptured. They then returned to me and asked me to enucleate, which I did. In several weeks the disease returned in the orbit, and in several more weeks the child died.

Case second I saw in Hustonville, in this state. I was there on professional business, and was consulted in reference to the growth, which had already broken through at the sclero-corneal junction. I diagnosed glioma of retina, and advised enucleation, which, after some debate, was submitted to. In enucleating the globe nearly all the orbital tissue was also removed. Notwithstanding this the disease soon returned and bulged between the lids. The surrounding glands were swollen and indurated. Death soon followed.

In neither of the cases was any regret expressed by the parents of the children that the operation had been done.

While in New York I assisted Dr. Agnew in enucleating an eye of a child for this same trouble. This case is a sadder as well as a more interesting one than the others. The parents had been married sixteen or eighteen years. This little boy, about eighteen months old, was their only child, and I think was the only one they ever had. In about six months after the first enucleation Dr. Agnew was compelled to remove the fellow eye for the same trouble. I heard from this case several years afterward, and there had been no return of the disease.

I forgot to say that the fellow eye of case second became blind several weeks before death, no doubt from glioma of retina.

The only means of diagnosing glioma of retina from sarcoma of choroid without the microscope is the age of patient. Glioma occurs only in children.

In review, then, an early enucleation in glioma of retina (or sarcoma of choroid) should always be advised. The possibility and probability of its returning, but in some internal organ, should be stated.—*Louisville Medical News.*

